# Clinical factors predicting risk for aspiration and respiratory aspiration among patients with Stroke^1^

**DOI:** 10.1590/0104-1169.0197.2545

**Published:** 2015-04-14

**Authors:** Ana Railka de Souza Oliveira, Alice Gabrielle de Sousa Costa, Huana Carolina Cândido Morais, Tahissa Frota Cavalcante, Marcos Venícios de Oliveira Lopes, Thelma Leite de Araujo

**Affiliations:** 2 Post-doctoral fellow, Escola de Enfermagem de Ribeirão Preto, Universidade de São Paulo, WHO Collaborating Centre for Nursing Research Development, Ribeirão Preto, SP, Brazil. Scholarship holder from Conselho Nacional de Desenvolvimento Científico e Tecnológico (CNPq), Brazil; 3 PhD, Professor, Faculdade Integrada da Grande Fortaleza, Fortaleza, CE, Brazil; 4 Doctoral student, Universidade Federal do Ceará, Fortaleza, CE, Brazil. Assistant Professor, Faculdade Católica Rainha do Sertão, Quixadá, CE, Brazil; 5 PhD, Professor, Departamento de Enfermagem, Universidade da Integração Internacional da Lusofonia Afro-Brasileira, Redenção, CE, Brazil; 6 PhD, Associate Professor, Departamento de Enfermagem, Universidade Federal do Ceará, Fortaleza, CE, Brazil; 7 PhD, Full Professor, Departamento de Enfermagem, Universidade Federal do Ceará, Fortaleza, CE, Brazil

**Keywords:** Nursing, Nursing Diagnosis, Respiratory Aspiration, Stroke, Nursing Assessment

## Abstract

**Objective:**

to investigate the association of risk factors with the Risk for aspiration nursing diagnosis and respiratory aspiration.

**Method:**

cross-sectional study assessing 105 patients with stroke. The instrument used to collect data addressing sociodemographic information, clinical variables and risk factors for Risk for aspiration. The clinical judgments of three expert RNs were used to establish the diagnosis. The relationship between variables and strength of association using Odds Ratio (OR) was verified both in regard to Risk for aspiration and respiratory aspiration.

**Results:**

risk for aspiration was present in 34.3% of the patients and aspiration in 30.5%. The following stood out among the risk factors: Dysphagia, Impaired or absent gag reflex, Neurological disorders, and Impaired physical mobility, all of which were statistically associated with Risk for aspiration. Note that patients who develop such a diagnosis were seven times more likely to develop respiratory aspiration.

**Conclusion:**

dysphagia, Impaired or absent gag reflex were the best predictors both for Risk for aspiration and respiratory aspiration.

## Introduction

Every year, 795 thousands people experience a new or recurrent episode of cerebrovascular accident (CVA) or stroke. Even though CVA is the second-leading cause of death around the world and is associated with the main cause of disability in many countries, the mortality rate decreased 34.8% from 1998 to 2008 and the real number of deaths caused by CVA decreased by 19.4%^(^ 1 ^)^. 

Despite this decreased rate, patients may experience many complications with the potential to affect mobility, speech and swallowing; swallowing disorders have a prevalence from 18 to 81% in the acute phase of stroke and may persist up to six months after the event, which can lead to a worse prognosis^(^ 2 ^-^ 3 ^)^.

Aspiration is the most severe aspect of dysphagia, defined as the entry of solids or fluids into airways below the vocal cords; it is more frequently observed when swallowing fluids^(^ 4 ^-^ 5 ^)^. It is worth noting that the concept of respiratory aspiration is well-defined in the health field, thus its prevention requires the joint efforts of a multidisciplinary team caring for a CVA patient. This situation shows the need to focus on the assessment of the Risk for aspiration nursing diagnosis in order to reduce complications that may arise from a stroke. 

According to NANDA International, Inc. (NANDA-I), the nursing diagnosis Risk for aspiration is defined as "the risk for entry of gastrointestinal secretions, oropharyngeal secretions, solids or fluids into the tracheobronchial passages"^(^ 6 ^)^. After the clinical validation process of this diagnosis in CVA patients, the risk factors found were: dysphagia; impaired or absent cough reflex; neurological disorders; use of gastrointestinal tubes; impaired physical mobility; impaired or absent gag reflex; and low headboard^(^ 7 ^)^. 

Therefore, improved understanding of the Risk for aspiration nursing diagnosis and the development of respiratory aspiration among patients with stroke enables identifying some needs and frailties to which nurses should pay attention, elect priorities, and upon which focus their work. Even though there are studies addressing respiratory aspiration, only one study^(^ 7 ^)^ was found that investigated this nursing diagnosis and its relationship with patients affected by CVA. 

Additionally, note that knowledge of associated factors is essential to rapidly establish these phenomena, since the methods considered to be the gold standard for diagnosing this condition, such as videofluroscopy or even endoscopy by optical fiber, are expensive and not widely available in healthcare facilities^(^ 8 ^)^. Hence, healthcare providers working with this population should be aware of this relationship so they can rapidly implement preventive measures. 

This study's aim was to investigate the association of risk factors with the Risk for aspiration nursing diagnosis and the development of respiratory aspiration among patients with stroke. 

## Method

Descriptive, cross-sectional study conducted with inpatients of a unit specifically providing care to CVA patients of a tertiary general public hospital, for referral for the treatment of this pathology, located in the Northeast, Fortaleza, CE, Brazil from March to August, 2013.

The Stroke unit is a multidisciplinary clinical care unit with 20 beds, dedicated to providing care to patients affected by CVA during the acute phase and complies with recommendations of Decree No. 665, April 12 2012, which regulates hospital facilities such as Emergency Rooms for CVA patients. 

The population consisted of patients with a medical diagnosis of CVA or transient ischemic attack admitted in the unit. The convenience sample was composed of 105 inpatients who met the following inclusion criteria: being 18 years old or older, being conscious, and able to obey to commands according to clinical assessment performed by the primary author. Discontinuation criteria were: a) having some clinical instability with risk of death and b) being transferred to another unit or healthcare facility, or being discharged. No patient was excluded from the study. 

Data were collected using a form based on the risk factors of the nursing diagnosis under study, which had been validated for CVA patients in a previous study^(^ 7 ^)^. Additionally, sociodemographic (sex, education, marital status, and age) and clinical variables (comorbidities, use of vasoactive drugs, sedative, oxygen therapy, type of CVA, number of episodes, and time since the event) were also verified. 

Data concerning the risk factors for the Risk for aspiration diagnosis were collected by two RNs with clinical experience of at least one year with CVA patients or critical patients. These RNs received prior training of 16 hours provided by the researcher, who included information concerning CVA, respiratory aspiration, nursing diagnoses, and Risk for aspiration. These professionals had a list with risk factors and their conceptual and operational definitions. Potential disagreements during data collection were discussed together with the researcher so that the pair could reach consensus. 

After a preliminary assessment, the presence of respiratory aspiration was verified using a test proposed by Daniels et al.^(9-10) ^that identified six clinical features: dysphonia, dysarthria, abnormal gag reflex, abnormal voluntary cough, cough after swallowing, and change of voice after swallowing. Aspiration was confirmed when at least two of the six clinical features were observed. This clinical assessment was performed due to the unavailability of a videofluroscopy (the gold standard) to confirm respiratory aspiration. Note that studies^(^ 9 ^-^ 10 ^)^ that established these clinical signs presented specificity at 89% for the detection of respiratory aspiration when compared to the gold standard.

Afterwards, data concerning this assessment, without the information of whether there was the presence of respiratory aspiration or not, was submitted to three judges, who met the following criteria: having a Master's degree; academic and professional background in the field of nursing diagnoses; and having provided care to patients with CVA or critical patients. The purpose of having these experts was to avoid the influence of data collection in terms of diagnostic inference, because these professionals were not aware of which of the patients had experienced respiratory aspiration during hospitalization. 

The judges received the instrument completed by the nurses during collection of each patient's data, presenting sociodemographic data, clinical variables, and risk factors for aspiration. The diagnosis under study was reported by most RNs. Hence, we decided to use the agreement reached by the experts to be the gold standard to identify the presence of this diagnosis, minimizing bias, considering the individual opinion of experts, as was performed in a prior study verifying accuracy^(^ 11 ^)^.

The results were organized in Excel 8.0 spreadsheets and analyzed using SPSS, version 20.0. The sample is descriptively presented according to sociodemographic and clinical data. Numerical variables are represented in terms of central tendency and dispersion. The Kolmogorov-Smirnov test was used to verify the numerical data's normality/symmetry.

Pearson's Chi-square was used to analyze association among data in the occurrence of expected frequencies above five and below 20 and organized into 2x2 tables; Fisher's exact test was used when expected frequencies were below five. Odds Ratio (OR) was used to verify strength of association. OR was computed in the cells with frequency equal to zero. A level of significance of 5% was adopted.

Ethical guidelines concerning research involving human subjects were complied with. Data were collected after approval was obtained from the Institutional Review Board at the Federal University of Ceará (protocol No. 215.770).

## Results

A total of 105 patients affected with CVA participated in this study. Most (54.3%) were men, had a partner (67.6%), were aged 57.84 years on average (± 14.48), were not currently employed (57.1%), and had studied 4.66 years on average (± 4.62). 

In regard to the type of stroke, ischemic events (88.6%) stood out, followed by transient ischemic attack (7.6%), and hemorrhagic events (3.8%). In regard to the recurrence of CVA, 68.6% had experienced more than one episode. The average duration of hospitalization was 142.62 hours (±108.96). The variables under study (age, education and time since CVA) presented asymmetric distribution (p value <0.05). 

In regard to the characterization of patients, no patient was using vasoactive or sedative drugs during the assessment and none were receiving low- or high-flow oxygen therapy. The following comorbidities were found: hypertension (63.8%), diabetes mellitus (24.8%), heart diseases (14.3%), and dyslipidemia (13.3%). 

The most frequent risk factors were Impaired or absent gag reflex (32.4%), followed by Impaired or absent cough reflex (28.6%), and Dysphagia (23.8%). In regard to the nursing diagnosis, the judges deemed that 34.3% of the patients affected by CVA presented the Risk for aspiration diagnosis, while 30.5% developed respiratory aspiration, which was confirmed by clinical assessment (Table 1). Additionally, most patients with CVA did not present risk factors for Risk for aspiration (31.4%) or one factor (29.5%), followed by those with two risk factors (16.2%), three (12.4%), four (7.6%), five (1.9%), and six factors (0.9%). 

**Table 1 -:** Risk factors for the diagnosis Risk for respiratory aspirations found among patients with stroke. Fortaleza, CE, Brazil, 2013

Risk factors		N	%
Impaired or decreased gag reflex		34	32.4
Impaired or absent cough reflex		30	28.6
Dysphagia		25	23.8
Neurological disorders		23	21.9
Impaired physical mobility		23	21.9
Low headboard		14	13.3
Use of gastrointestinal tubes		3	2.9
	Nursing diagnosis		
Risk for aspiration		36	34.3
Respiratory aspiration		32	30.5

Association (Odds Ratio) between risk factors and the Risk for aspiration nursing diagnosis was stronger for the following clinical features: Impaired or absent gag reflex (OR 19.825), Dysphagia (OR 4.214), and Impaired physical mobility (OR 2.636). Note that the individuals with neurological disorders were 87% less likely to develop Risk for aspiration (OR 0.134) (Table 2). 

**Table 2 -:** Estimates of association of risk factors for the occurrence of the Risk for aspiration nursing diagnosis among patients with stroke. Fortaleza, CE, Brazil, 2013

Variable		Risk for aspiration		Total	p*	OR^†^	IC 95%^‡^
		Present	Absent				
Dysphagia					˂0.002	4.214	1.642-10.815
	Present	15	10	25			
	Absent	21	59	80			
	Total	36	69	105			
Impaired or absent cough reflex					0.560	0.762	0.306-1.897
	Present	9	21	30			
	Absent	27	48	75			
	Total	36	69	105			
Neurological disorders					0.004	0.134	0.030-0.612
	Present	2	21	23			
	Absent	34	48	82			
	Total	36	69	105			
Use of gastrointestinal tubes					˂0.972	0.957	0.084-10.927
	Present	1	2	3			
	Absent	35	67	102			
	Total	36	69	105			
Impaired physical mobility					0.042	2.636	1.023-6.792
	Present	12	11	23			
	Absent	24	58	82			
	Total	36	69	105			
Impaired or absent gag reflex					0.001	19.825	7.029-55.913
	Present	26	8	34			
	Absent	10	61	71			
	Total	36	69	105			
Low headboard					0.630	0.738	0.214-2.540
	Present	4	10	14			
	Absent	32	59	91			
	Total	36	69	105			

* Fisher's exact test/

† OR: Odds Ratio/

‡ CI: Confidence Interval

In addition to associations presented in Table 2, we also found association between Risk for aspiration and the presence of one risk factor (p<0.001) and when the patients were assessed within 72 hours of the event (p<0.048). In the first situation, patients were 4.3 times more likely to present Risk for aspiration, regardless of the risk factor (OR 4.307). In the second situation, patients were 59% less likely to develop Risk for aspiration when the CVA episode had occurred within 72 hours (OR 0.419). Note that no association was found between the nursing diagnosis and the recurrence of episodes of CVA (p<0.562) or when a different quantity of risk factors were found (two, three, four, five or six factors) or with different intervals of time since the event (24 hours, 48 hours, 96 hours, 120 hours or more than 120 hours). 

When associating risk factors with the presence of respiratory aspiration, association was stronger with Dysphagia (OR 12.122), Impaired or absent gag reflex (OR 11.183), and with Impaired physical mobility (OR 2.262). Patients with the Risk for aspiration nursing diagnosis were seven times more likely to develop respiratory aspiration (OR 7.381) (Table 3). 

**Table 3 -:** Estimates of association of risk factors for the occurrence of Respiratory aspiration among patients with stroke. Fortaleza, CE, Brazil, 2013

Variables		Respiratory aspiration		Total	p*	OR^†^	IC 95%^‡^
		Present	Absent				
Dysphagia					<0.001	12.122	4.258-34.515
	Present	18	7	25			
	Absent	14	66	80			
	Total	32	73	105			
Impaired or absent cough reflex					0.689	1.205	0.486-2.985
	Present	10	20	30			
	Absent	22	53	75			
	Total	32	73	105			
Neurological disorders					0.996	0.998	0.365-2.725
	Present	7	16	23			
	Absent	25	57	82			
	Total	32	73	105			
Use of gastrointestinal tubes					0.169	4.800	0.419-54.958
	Present	2	1	3			
	Absent	30	72	102			
	Total	32	73	105			
Impaired physical mobility					<0.042	2.262	1.023-6.931
	Present	11	12	23			
	Absent	21	61	82			
	Total	32	73	105			
Impaired or absent gag reflex					<0.001	11.183	4.237-29.516
	Present	22	12	34			
	Absent	10	61	71			
	Total	32	73	105			
Low headboard					<0.649	1.317	0.404-4.295
	Present	5	9	14			
	Absent	27	64	91			
	Total	32	73	105			
Risk for respiratory aspiration diagnosis					<0.001	7.381	2.928-18.604
	Present	21	15	36			
	Absent	11	58	69			
	Total	32	73	105			

* Fisher's exact test

† Odds Ratio

‡ Confidence Interval

Association between the frequency of risk factors and the occurrence of respiratory aspiration was statistically significant with the presence of two risk factors (p<0.004), three (p<0.001), and four risk factors (p<0.004). In these situations, the patients were respectively three (OR 3.388), four (OR 4.383) and six times (OR 6.533) more likely to develop respiratory aspiration. No significant association was found between time since the event (24 hours, 48 hours, 72 hours, 96 hours, 120 hours and more than 120 hours) or recurrence of stroke with the development of respiratory aspiration.

## Discussion

In general, the sociodemographic profile of the 105 patients with CVA assessed in this study was similar to that reported by international studies, i.e., mostly men, married, with a low level of education, and not currently employed^(^ 12 ^-^ 14 ^)^. 

Note that age may influence swallowing because many changes occur in the masticatory apparatus (e.g., lips, tongue, cheeks), which may change mastication, swallowing, breathing, voice, speech and increase the risk for respiratory aspiration^(^ 15 ^-^ 17 ^)^. Additionally, such changes may also gradually decrease oropharyngeal and laryngeal sensitivity, decrease or eliminate swallowing reflex, or delay oral transit and swallowing^(^ 18 ^)^.

Most participants lived with a partner (67.6%), which is important because a caregiver, often the wife, generally appears in family relationships and helps the individual impaired due to a CVA^(^ 19 ^)^. Therefore, it is essential that the caregiver be trained by nurses to recognize the risk factors for respiratory aspiration, because it contributes to malnutrition, dehydration, and respiratory infections, among other problems that worsen the patient's condition. 

In regard to the characterization of CVA, ischemic events stood out. One study conducted in the USA assessed 333,865 inpatients with CVA from 2001 and 2007 and reports that 82.4% of the patients presented an ischemic stroke while mortality was higher for those with hemorrhagic events (27.2%)^(^ 20 ^)^. Another study reports that ischemic stroke were more common, though hemorrhagic events most frequently resulted in negative changes in swallowing^(^ 21 ^)^. 

When the occurrence of comorbidities was investigated, hypertension and diabetes mellitus stood out. The INTERSTROKE study (a multicenter study involving 36 countries that identified risk factors among patients who experienced their first episode of CVA) reports that these clinical conditions, history of hypertension (OR 3.89) and diabetes mellitus (OR 1.36), increase the risk of a CVA^(^ 12 ^)^. 

In regard to the Risk for aspiration nursing diagnosis and respiratory aspiration, this study's results are similar to those reported by another study^(^ 7 ^)^ that assessed 24 patients in the acute phase of stroke. The study reports a prevalence of 58.3% for the Risk for aspiration diagnosis and 37.5% of the patients developed respiratory aspiration after 72 hours. Note that the occurrence of silent aspiration was not verified, which may have decreased the prevalence of the phenomenon in the studied population.

The incidence of respiratory aspiration among patients affected by a stroke is approximately 50% and about half of those patients experience silent aspiration^(^ 5 ^,^ 21 ^)^. Even though the prevalence found in this study was lower, it does not diminish the clinical relevance of this problem because there is a clear relationship between respiratory aspiration and an increased number of complications, longer hospitalizations, higher mortality rates, higher hospital costs, and more frequent use of gastrostomy feeding tubes.

Authors state that the assessment of patients with CVA to verify whether they present aspiration should be performed early, within 72 hours at most from the beginning of treatment in order to identify patients at risk and plan care to be implemented^(^ 22 ^)^. This study's participants assessed within 72 hours were less likely to develop Risk for aspiration, corroborating these findings. 

In regard to the number of risk factors, we observed that the presence of one factor increased the likelihood of developing Risk for aspiration and the presence of two or more factors increased the likelihood of respiratory aspiration. Studies associating the number of risk factors with the development of these phenomena were not found; however, many studies show the relevance of these factors to the development of both Risk for aspiration^(^ 7 ^)^ and respiratory aspiration^(^ 4 ^-^ 5 ^,^ 21 ^-^ 22 ^)^. 

Risk for aspiration and the presence of respiratory aspiration were assessed simultaneously and the following factors contribute to these clinical conditions: Dysphagia, Impaired or absent gag reflex, and Impaired physical mobility. In this study, these were good predictors for both phenomena.

Note that in this study we found an association between the risk factor Neurological disorders and the nursing diagnosis Risk for aspiration. This risk factor, however, behaved as a protective factor. This may have been a spurious result since the literature shows that the type and localization of CVA, as well as the existence of another neurological disease, may impact the swallowing process and lead to respiratory aspiration^(^ 4 ^,^ 23 ^)^. According to conceptual analysis, these disorders are linked to the stroke severity (hemorrhagic, bilateral and/or brainstem), may or may not be associated with diseases and prior conditions such as brain injury or Alzheimer's^(^ 7 ^,^ 18 ^,^ 24 ^)^.

Concerning the relationship of dysphagia and respiratory aspiration, one study reports an increase of 22 times in the likelihood of developing aspiration when dysphagia was present (OR 21.83) at the time of clinical assessment and an increase of 10 times when the exam was performed by videofluroscopy (OR 10.50)^(^ 25 ^)^. Assessment of the relationship between dysphagia and Risk for aspiration showed that dysphagia is accurate in determining the diagnosis (specificity at 88.8% and a positive predictive value of 90.9%)^(^ 7 ^)^. Therefore, an early assessment of this clinical indicator is essential so that nurses can detect clinical signs, or through screening results, to investigate for swallowing difficulties.

Dysphagia is very common among patients who suffered a stroke. Statistical differences concerning its prevalence depend of the diagnostic method used, time since the event occurred, and site of the lesion. This event contributes to the use of enteral feeding tubes, which increase the duration of hospitalizations^(5) ^and interfere in the patients' independence to feed him/herself and perform activities of daily living^(^ 15 ^)^.

Another factor that may lead patients with CVA to need enteral feeding support is a lack of gag reflex, which produces severe disruption of the reflex of the pharyngeal phase of swallowing, resulting in persistent dysphagia^(^ 3 ^)^. This situation also contributes to respiratory aspiration and aspiration pneumonia, which is evidenced by a persistent cough with sputum or by other signs such as fever, tachypnea, or focal consolidation confirmed by radiographic imaging^(^ 23 ^)^.

Impaired physical mobility was observed in 21.9% of the patients. Even though it is not listed in NANDA-I for the diagnosis under study, Impaired physical mobility was proposed in a clinical validation study and presented good accuracy (sensitivity at 80%, specificity at 77.7%, a positive predictive value of 85.7% and a negative predicative value of 70%)^(^ 7 ^)^. In this study, this risk factor doubled the likelihood of patients presenting Risk for aspiration and developing respiratory aspiration. 

Even though the other risk factors observed in the population under study were not associated with the diagnosis or with the development of respiratory aspiration, much has been discussed in the literature about association with these events; thus, it is essential that nurses continuously assess these risk factors^(^ 5 ^,^ 7 ^,^ 9 ^)^. 

Finally, the results presented reinforce the importance of clinically assessing these patients with a focus on swallowing, as well as a respiratory assessment to provide early identification of the factors that have been shown to be the best predictors for the phenomena, or the consequences of these phenomena, in order to favor accuracy of nurses' diagnostic process.

## Conclusion

The risk factors that better predicted the phenomena under study include: Impaired or decreased gag reflex; Dysphagia; and Impaired physical mobility. Additionally, the patients with the diagnosis Risk for aspiration were at an increased risk of developing respiratory aspiration. 

The considerable prevalence of this diagnosis shows the need to implement nursing interventions, such as educational activities, while the patient remains in the hospital environment and also upon hospital discharge, so that patients and caregivers can acquire all the knowledge required to continue delivering care at home.

This study's limitations include the fact that, even though the pair of nurses collecting data received the same training, bias may exist nonetheless. Selection bias also is possible, since many patients presented respiratory aspiration, whether due to the patient's condition or due to the dynamics of the sector's multidisciplinary care. Finally, because this is a cross-sectional study, causality cannot be established.

Given this study's findings, further studies addressing this diagnosis are needed so that nurses can recognize clinical evidence and characteristics and devise more specific care plans, enabling more effective care. Therefore, further studies seeking the predisposing factors for Risk for aspiration and respiratory aspiration in other clinical situations and among patients in different phases of stroke are required, because this condition may persist even during rehabilitation. 
